# Supplementary material for: TMO5 regulates PIN1 polarity convergence and organogenesis downstream of MONOPTEROS in the Arabidopsis shoot
Source: Development. 2025 Dec 12;152(24):dev205255. doi: 10.1242/dev.205255 (PMC12752488; doi:10.1242/dev.205255)
Supplement: Supplementary information [file develop-152-205255-s1.pdf]

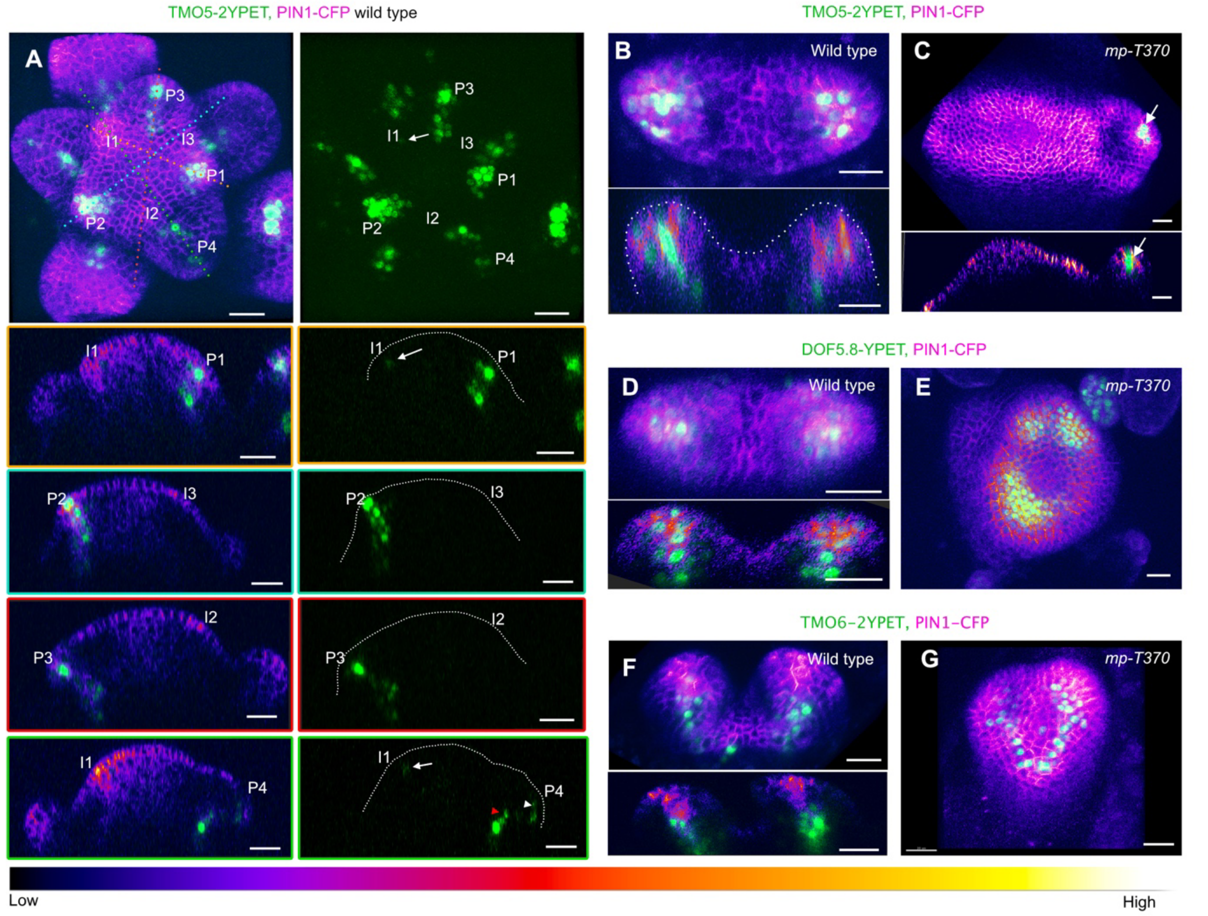

Fig. S1. Expression pattern of TMO5, DOF5.8, and TMO6 in wild type and *mp* mutant meristems. (A) TMO5::TMO5-2YPET expression (green) in wild type inflorescence meristem partially overlapping with PIN1::PIN1-CFP (intensity-based colouring; the look-up table displays the signal intensity range) at various organ initiation stages. Incipient primordia (i3, i2, i1) and developing primordia (p1, p2, p3, p4) are labeled. Right panels show the green channel from the merged images (n= 13). (B) TMO5::TMO5-2YPET expression overlapping with PIN1::PIN1-CFP in wild type vegetative meristem. Lower panel shows longitudinal optical section (n= 21) (C) TMO5::TMO5-2YPET expression (arrow) in the rarely formed leaves of the *mp-T370* mutant dome meristem. Lower panel shows longitudinal optical section (n= 19). (D) DOF5.8::DOF5.8-YPET expression (green) wild type vegetative meristem. Lower panel shows longitudinal optical section (n= 11). (E) DOF5.8::DOF5.8-YPET expression in *mpT370* mutant pin-like meristem, showing reduced expression at later stages (n= 15). (F) TMO6::TMO6-2YPET expression (green) in wild type vegetative meristem. Lower panel shows longitudinal optical section (n= 13). (G) TMO6::TMO6-2YPET expression in *mp-T370* mutant pin-like meristem (n= 13). Scale bar: 20 μm.

Development • Supplementary information

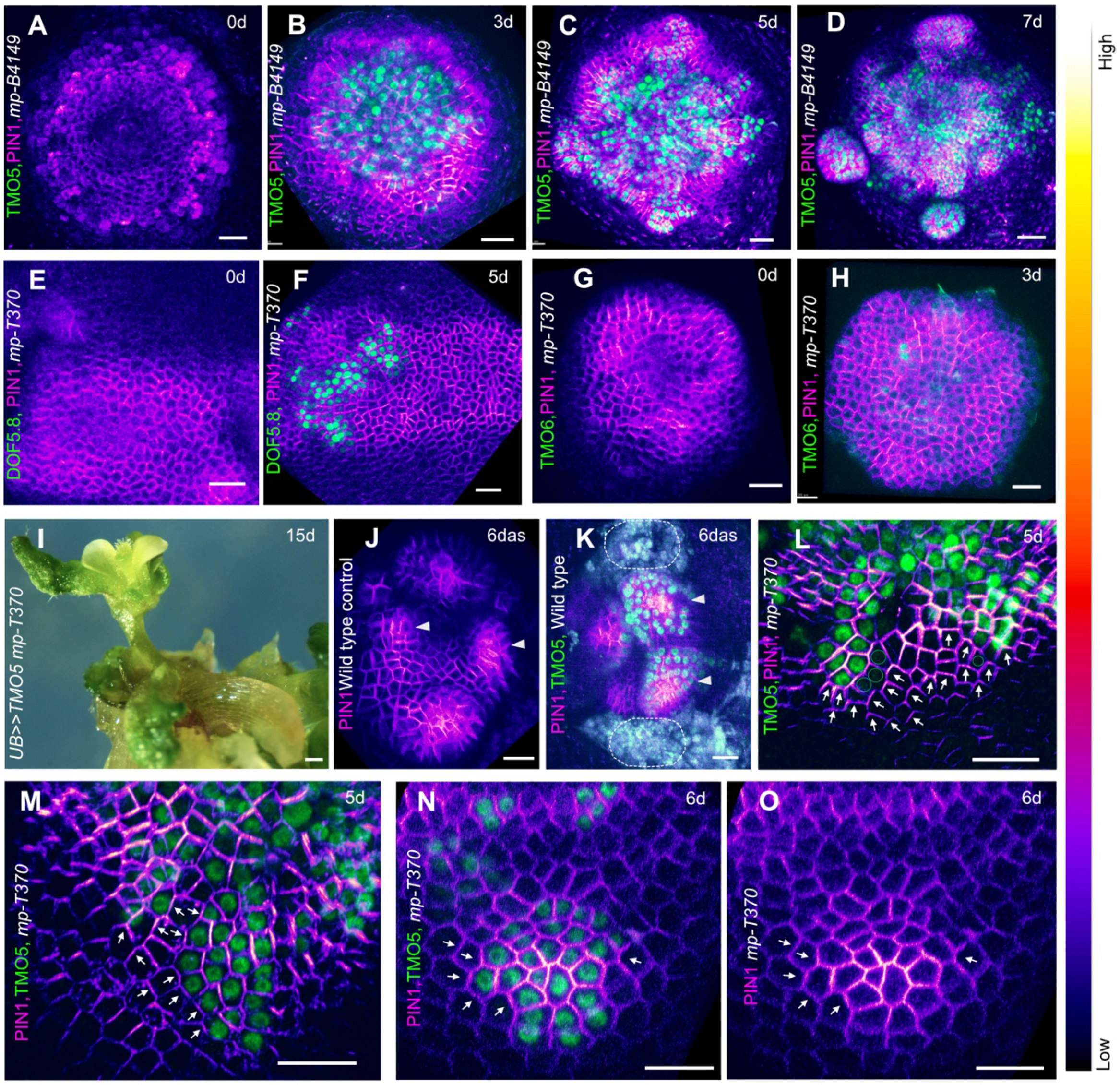

Fig. S2. **TMO5 promotes PIN1 polarization and organogenesis.** (A-D) Activation of PIN1-GFP expression (intensity-based colouring; the look-up table displays the signal intensity range), convergence formation, and organ outgrowth in *mp* mutant shoot apical meristems following local activation of TMO5-2YPET clones (green) (n=14). (E-H) No activation of PIN1-CFP expression or organ outgrowth in *mp* mutant after local expression of clones of DOF5.8-YPET (E,F; n=12) or TMO6-YPET (G,H; n=15). (I) Rescue of the *mp* mutant flower phenotype by transient ubiquitous expression of TMO5-2YPET with 8 days of Dex treatment, followed by 7 days without Dex (n= 5). (J) Six-day-old wild type vegetative meristem with PIN1-GFP expression, showing four leaves. Arrowheads indicate the third and fourth leaves (n= 12). (K) Six-day-old wild type vegetative meristem with PIN1-GFP expression, showing altered positioning of the third and fourth leaves (arrowheads) following clonal activation of TMO5-2YPET (green). The first and second leaves were removed (dotted circles) to visualize the repositioned organs (n= 7). (L-M) PIN1-CFP polarization in neighboring cells toward TMO5-expressing clones in *mp* mutant meristems. Arrows indicate polarity; green circles in (L) mark weakly detectable TMO5 clones (n= 17). (N-O) PIN1-CFP convergence within TMO5-expressing clones and polarization in neighboring cells in *mp* mutant meristems, with (O) showing a separate PIN1-CFP channel for clarity (n= 24). Scale bar: 20 µm, except for (I) where it is 1 mm.

Development • Supplementary information

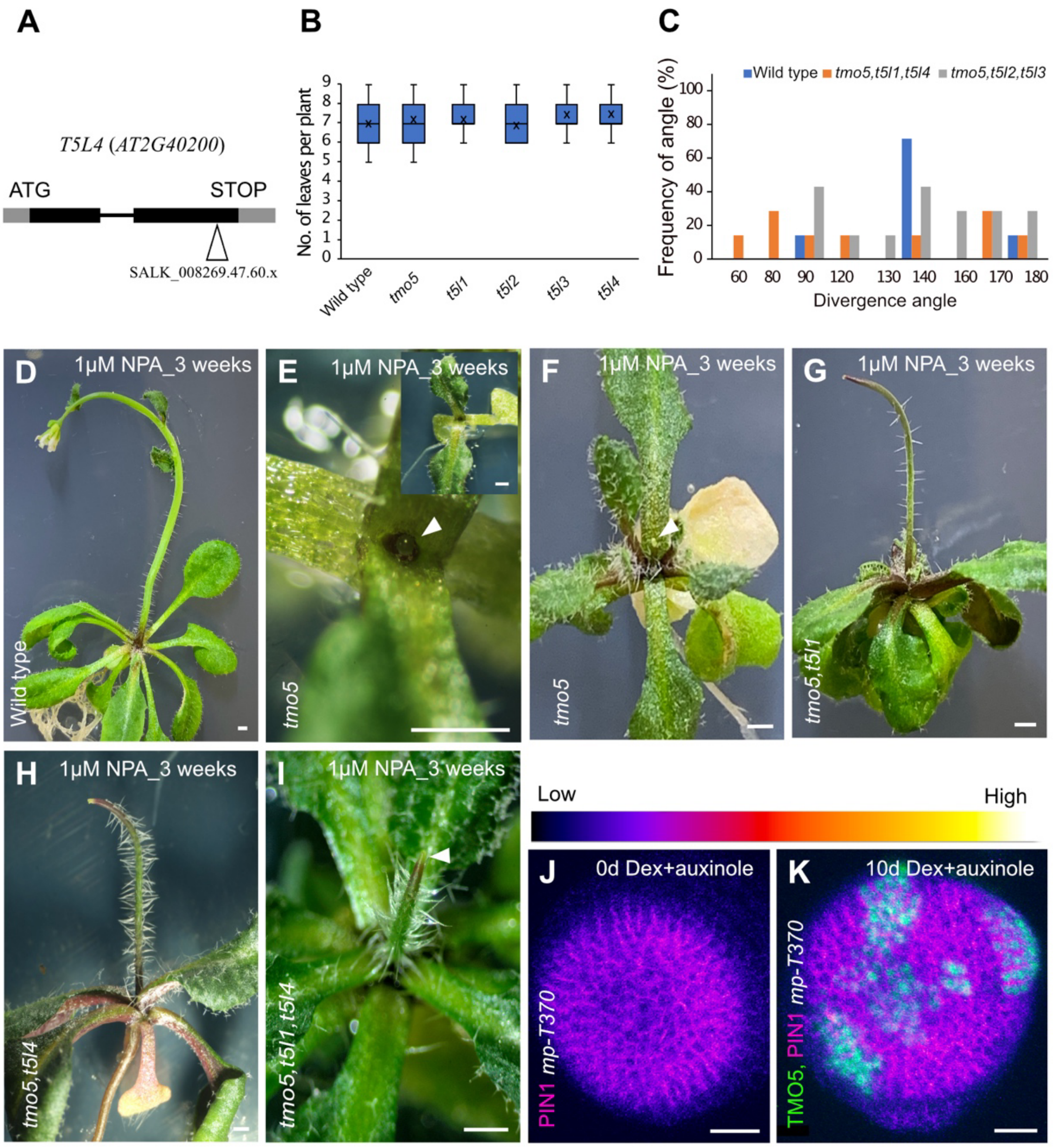

Figure S3. ***TMO5* and *TMO5-LIKE* genes contribute to organ formation.** (A) Gene structure and location of T-DNA insertion of *t5l4* mutant (AT2G40200, SALK_008269.47.60, N508269) (B) Leaf formation in wild type and *tmo5/t5l* mutants. (n=31 plants per genotype; poisson regression; box plot shows median (central line), mean (cross), first and third quartiles (top and bottom edges), and minimum and maximum values excluding outliers (whiskers)). (C) Phyllotaxis in *tmo5, t5l1, t5l4* and *t mo5, t5l2, t5l3* triple mutants, measured as the divergence angle between adjacent siliques in the inflorescence, compared to wild type (most frequent angle 137°; n= 7-14 plants). (D) Wild-type inflorescence with flowers after 1 µM NPA treatment. (E–I) Pin-like inflorescences in *tmo5* single, *tmo5,t5l1* and *tmo5,t5l4* double, and t*mo5,t5l1,t5l4* triple mutants after 1 µM NPA treatment. Arrowheads indicate dome meristem in (E) and pin-like inflorescences in (F and I). Inset in (E) shows an overview image. (J–K) *mp* mutant meristem treated with 100 µM auxinole for 0d and 10 d after TMO5-2YPET (green) clonal activation, showing no PIN1-CFP convergence (intensity-based coloring; look-up table shows signal range) and suppressed organ formation (n= 11), in contrast to the mock control shown in (Fig. 3P,Q). Scale bar: 1 mm in (D-I); 20 µm in (J–K).

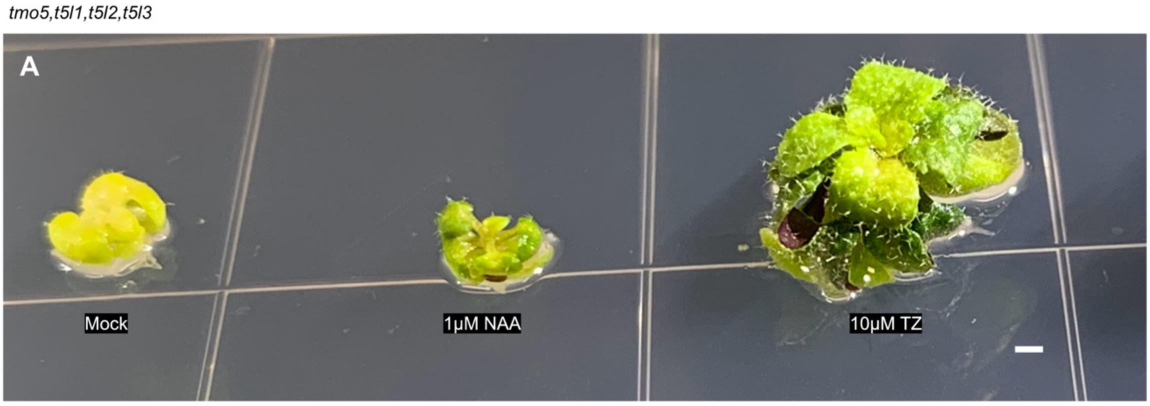

Fig. S4. **Cytokinin partially rescues leaf formation in *tmo5* quadruple mutant** (A) Leaf formation in the *tmo5,t5l1,t5l2,t5l3* quadruple mutant after treatment with cytokinin (10 µM TZ) with no effect from auxin (1 µM NAA) alone. Scale bar: 1 mm.

Table S1. List of oligos used in this study.

Primer	Sequence
Primers for generation of sectoring constructs:	
DOF5.8-forward	agatctCAACATGCCTTCTGAATTCAGTGAATC
DOF5.8-reverse	agatctccCGCTACGTAGTCTCCAGACACGA
TMO5-forward	ggatccaaAATGTACGCAATGAAAGAAGAAGACTG
TMO5-reverse	agatctccATTATAACATCGATTCACCATCTTACTAG
TMO6-forward	cggcccaacaggatcGAAGCAATGGGACTATCATATTCATCA
TMO6-reverse	agccgcagcaggatccgcCATTAAAGCACCAGAATTAATGTAGTTC
Primers for generation of translational reporters:	
DOF5.8g-forward	ctcgagGGACCCATGAAAGCTTCTTTCTTTGCT
DOF5.8g-reverse	agatctccCGCTACGTAGTCTCCAGACACGA
TMO5g-forward	ctcgagGGAGAAAGAGCCCAGAATGTTGAACGT
TMO5g-reverse	ggatccgcATTATAACATCGATTCACCATCTTACTAG
TMO6g-forward	ctcgagCCAATCACTCTGTCGTTGGTTCCGTT
TMO6g-reverse	ggatccgcCATTAAAGCACCAGAATTAATGTAGTTC
T5L1g-forward	ctcgaggaacataggtccaaagtccgtctttagtc
T5L1g-reverse	ggatccgcCCTCTGATTATATTGTTGTTGTTGTTCGACG
T5L2g-forward	ctcgagggttcttgcgtctgaattagcaacc
T5L2g-reverse	ggatccgcGACCATTATGATGTGATCCAGCGCACGA
T5L3g-forward	ctcgagTCATTGTTCATGTTATTGAC
T5L3g-reverse	ggatccgcCACCATTATGATGTGATCCAGCGCACGA
T5L4g-forward	ctcgagtggacaacacatagcctgga
T5L4g-reverse	ggatccgcTTCATTGTGAGAATAATGAGAAGAGA
Primers for genotyping	
tmo5 forward	TGATCCTTCAAAATTTCTCTTTATTTAG
tmo5 reverse	CAAACCCAAGCTCTATACATTTCC
t5l1 forward	ctccacatatcctgcaaaccattgg
t5l1 reverse	GTGGTGTTGGGTAATATGCTACGGAGC
t5l2 forward	GCAAAAGTGGTTCAACGAGTC
t5l2 reverse	AAAGGCAGAACTCTTTAGGGG
t5l3 forward	ctccggcgactacatgacgactatg
t5l3 reverse	CTCCACCACCAGAACTACGTTCCATC
t5l4 forward	CTTTTTGCTGCGAAGATCAAC
t5l4 reverse	ttgagttggtcattttgtttcg
